# Advantages of 3D High-Resolution Vessel Wall Imaging in a Patient With Blood Blister-Like Aneurysm: A Case Report and Literature Review

**DOI:** 10.7759/cureus.58376

**Published:** 2024-04-16

**Authors:** Toru Otsuka, Kazufumi Kikuchi, Osamu Togao, Koji Yamashita, Soh Takagishi, Koichi Arimura, Akira Nakamizo, Kousei Ishigami

**Affiliations:** 1 Clinical Radiology, Kyushu University, Fukuoka, JPN; 2 Molecular Imaging and Diagnosis, Kyushu University, Fukuoka, JPN; 3 Neurosurgery, Kyushu University, Fukuoka, JPN

**Keywords:** internal carotid artery, subarachnoid hemorrhage, magnetic resonance imaging, vessel wall imaging, blood blister-like aneurysm

## Abstract

Blood blister-like aneurysms (BBAs) are rare and challenging intracranial aneurysms. They pose significant diagnostic and surgical risks due to their delicate walls. Accounting for a small percentage of intracranial aneurysms, BBAs are pathologically pseudoaneurysms, often resulting from arterial dissection, with a high tendency to rupture. This report underscores the critical nature of BBAs by reviewing a case in which subarachnoid hemorrhage caused by a BBA rupture was difficult to diagnose with conventional imaging. We highlight the efficacy of three-dimensional (3D) high-resolution vessel wall imaging (VWI) in discerning the subtle vascular abnormality of BBAs. The integration of the black-blood imaging technique within VWI provides superior contrast between the aneurysm and surrounding tissues, facilitating clearer visualization of the aneurysmal wall. The use of 3D T1-weighted imaging provides intricate details of the vessel wall including its contrast enhancement, which is crucial for a comprehensive assessment of a ruptured aneurysm. This case is consistent with the existing literature, supporting the role of VWI in the identification of ruptured BBAs, an area with limited but growing information on its diagnostic value. VWI is precise and accurate in the preoperative diagnosis of BBAs, emphasizing its potential to improve patient management and outcomes, especially in conditions with high risks of morbidity and mortality.

## Introduction

Blood blister-like aneurysms (BBAs) are rare aneurysms comprising 0.3-1.0% of intracranial aneurysms, 0.5-2.0% of ruptured intracranial aneurysms, and 0.9-6.5% of internal carotid artery (ICA) aneurysms [1-5]. They usually appear at the anteromedial or anterior wall of the supraclinoid segment of the ICA [1]. These aneurysms are characterized by small, hemispherical shapes and fragile walls, which pose significant risks for diagnosis and surgical intervention [3]. Pathologically, BBAs are pseudoaneurysms resulting from arterial dissection that are prone to rupture and can grow rapidly. Their fragile nature, typically with only a thin layer of adventitia or a blood clot, is associated with a risk of re-rupture. Despite significant advances in neuroimaging, radiological findings in conventional vascular imaging, such as computed tomography angiography (CTA), magnetic resonance angiography (MRA), and digital subtraction angiography (DSA), are sometimes indeterminate for BBAs. However, patients with BBAs tend to have a severe course because of the high rebleeding rate of BBAs. Therefore, early diagnosis and treatment of subarachnoid hemorrhage (SAH) due to rupture of BBAs may improve the clinical course of patients with BBAs.

Here, we present a case of SAH caused by a ruptured BBA for which conventional diagnostic methods were insufficient, but for which three-dimensional (3D) high-resolution vessel wall imaging (VWI) proved to be useful in diagnosing this condition. We discuss the imaging characteristics of BBAs and highlight the significance of VWI in the identification and diagnosis of this complex condition. Through this case study, we aim to underscore the potential of VWI in enhancing the diagnostic landscape for BBAs and improving patient outcomes.

## Case presentation

A woman in her 70s with hypertension and dyslipidemia who had undergone a right total knee arthroplasty experienced sudden and severe pain in her back and hip while taking a shower. She had a mild disturbance of consciousness and rapidly fell into a coma after generalized tonic convulsions. Her vital signs were stable, and her laboratory data showed no significant abnormalities. Initial CT scans at the presentation revealed an SAH. Her medical history was notable for hypertension, hyperlipidemia, and insomnia, and her father had a history of cerebral infarction. Moreover, on physical examination, her Glasgow Coma Scale was E4V5M6, indicating confusion without paralysis but compromised respiratory function necessitating bag-valve-mask ventilation due to low percutaneous oxygen saturation.

**Figure 1:**
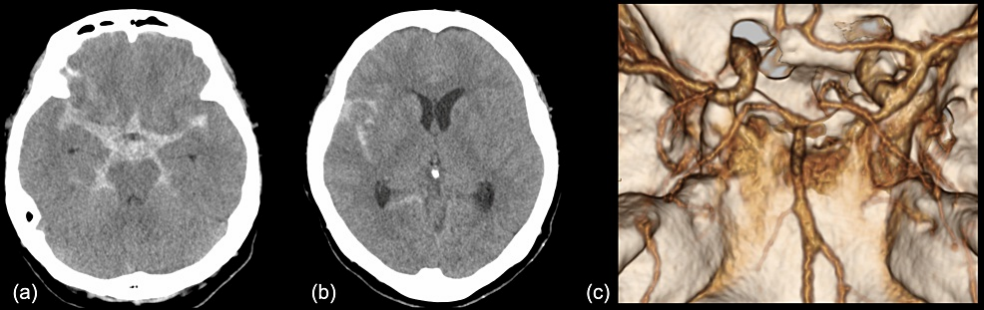
Initial head computed tomography (CT) Initial head computed tomography (CT) on the first day shows diffuse subarachnoid hemorrhage extending from the right Sylvian vallecula and fissure to the suprasellar cistern and mild hydrocephalus, classified as modified Fisher group 4 (a, b). Three-dimensional CT angiography shows no aneurysm (c).

Head CT demonstrated diffuse SAH extending from the right Sylvian vallecula and fissure to the suprasellar cistern, accompanied by mild hydrocephalus (Figures 1a-1b; classified as modified Fisher group 4). Subsequent 3D-CTA failed to reveal any conspicuous aneurysms (Figure 1c).

**Figure 2:**
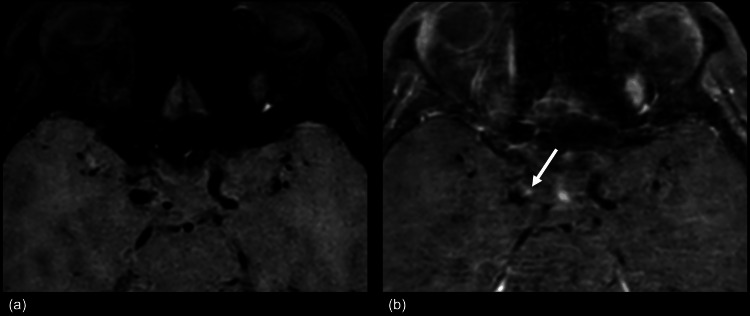
Contrast-enhanced vessel wall imaging Contrast-enhanced vessel wall imaging (VWI) shows spotty enhancement with a half-dome shape at the superior wall of the right supraclinoid internal carotid artery (Figure 2b) compared with a non-enhanced VWI (Figure 2a).

Subsequent magnetic resonance imaging (MRI; 3-tesla Ingenia Elition X 3.0 T; Philips Healthcare, Best, the Netherlands) using a 15-channel head coil was performed to identify the bleeding point of the aneurysm. The 3D turbo spin-echo imaging parameters for the VWI were repetition/echo time, 350/14 ms; flip angle, 90º; number of excitations, 1; matrix, 200 × 200 (recon. 512 × 512); field of view, 160 × 160 × 132 mm^3^; voxel size, 0.8 × 0.8 × 0.8 mm^3^ (recon. 0.3 × 0.3 × 0.4 mm^3^); turbo spin echo factor, 13; Compressed SENSE factor, 7; k-space filling direction, cartesian; motion-sensitized driven equilibrium for black-blood technique; velocity-encoding (VENC), 3 × 3 × 3 mm/s; and scan time, 4 min 36 s. We obtained the VWIs (Figures 2a-2b) before and after intravenous administration of gadobutrol (Gadovist; Bayer; 0.1 mmol/kg). The contrast-enhanced VWI showed spotty enhancement with a half-dome shape at the anterior wall of the right supraclinoid ICA (Figure 2b) compared with a non-enhanced image (Figure 2a).

**Figure 3:**
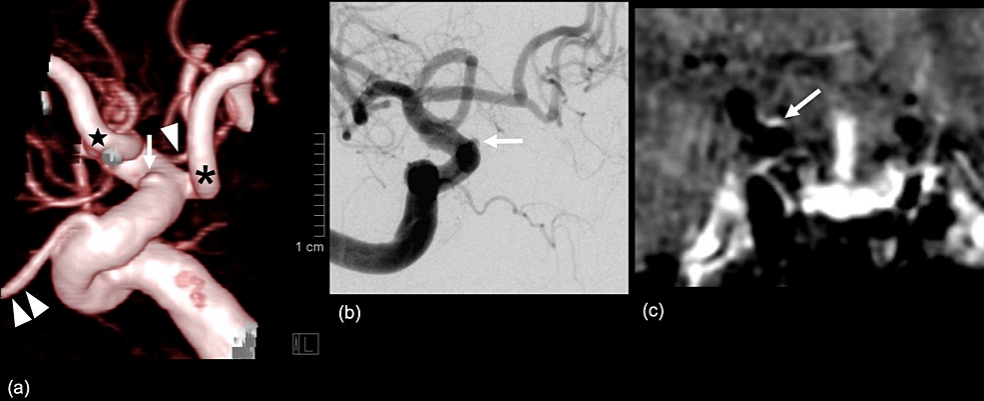
Digital subtraction angiography and contrast-enhanced vessel wall imaging The volume rendering image from the rotated four-dimensional digital subtraction angiography (DSA) shows a slight bulge (↓) on the superior wall of the supraclinoid portion of the right internal carotid artery (Figure 3a). Fine-tuned DSA (right anterior oblique 27º, caudal 9º) shows a hemispherical protrusion (Figure 3b). The corresponding reconstructed coronal-enhanced vessel wall imaging confirms an enhancement at the site of the bulge (Figure 3c), supporting the diagnosis of a blood blister-like aneurysm. ↓ blood blister-like aneurysm, (△) anterior choroidal artery, (△△) ophthalmic artery, (*) fetal-type posterior communicating aneurysm, (★) anterior cerebral artery

This spotty enhancement suggested an intramural hematoma caused by focal dissection of the vessel wall. DSA was performed the next day (day 2). Volume rendering image from the rotated 4D DSA showed a slight bulge on the superior wall of the supraclinoid portion of the right ICA (Figure 3a). The fine-tuned DSA showed a hemispherical protrusion (Figure 3b). The corresponding reconstructed coronal-enhanced VWI confirmed an enhancement at the site of the bulge (Figure 3c), supporting the diagnosis of a BBA.

**Figure 4:**
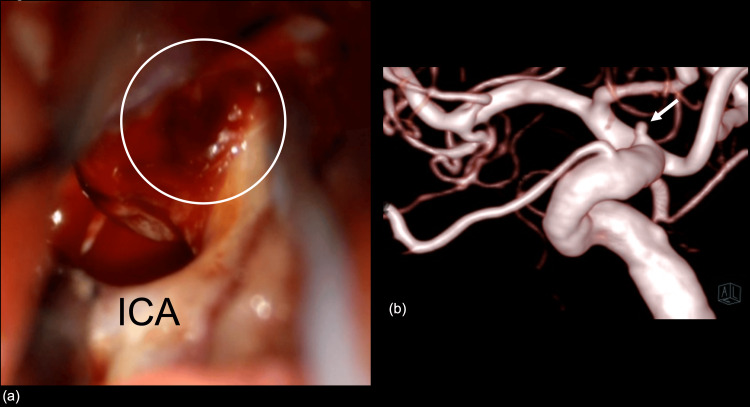
Intraoperative photograph and follow-up computed tomography angiography An intraoperative photograph shows the blood blister-like aneurysm on the right internal carotid artery (Figure 4a). The enlarged aneurysm was identified on the follow-up computed tomography angiography (Figure 4b), and the patient underwent endovascular treatment with stent-assisted coil embolization. ICA: internal carotid artery

On day 4, the patient underwent surgery for SAH due to a rupture of BBA on the right ICA. Although trapping of BBA after superficial temporal artery to middle cerebral artery bypass was initially planned according to the balloon Matas occlusion test, coating of BBA was finally performed instead of trapping due to the decrease of transcranial motor-evoked potential amplitude after temporal ICA occlusion despite the patency of superficial temporal artery to middle cerebral artery bypass. Intraoperative findings showed the BBA on the right ICA (Figure 4a). The follow-up DSA on day 11 identified the enlarged aneurysm (Figure 4b), and the patient underwent endovascular treatment with stent-assisted coil embolization. The procedure was successful; she had an uneventful course with no complications and was transferred to another hospital for rehabilitation.

## Discussion

This case report highlights the clinical significance of BBAs, which pose diagnostic challenges due to their small size, subtle presentation, and high risk of re-rupture associated with poor prognostic outcomes [1,6,7]. BBAs often manifest at non-branching sites of the ICA. They are typically small and thin-walled, and their hemispherical or conical shape may resemble other vascular abnormalities, particularly those associated with atherosclerosis [6]. These characteristics contribute to the difficulty of distinguishing BBAs from other types of aneurysms using standard imaging modalities, such as DSA and CTA, which often fail to accurately capture their distinct features [1,6,7].

Although the pathogenesis of BBAs is not fully understood, arterial dissection has been increasingly recognized as a contributing pathological process [4,8], often described as a pseudoaneurysm enveloped by adventitia [2,4,5]. Histologic studies have shown that BBAs involve lacerations at the border between arteriosclerotic and normal carotid walls because of the degeneration of the internal elastic lamina and the media [4,8] and loss of the internal elastic lamina, vascular intima, and media, sometimes appearing as only a fragile fibrous layer [2,4,9-12]. Additionally, inflammatory cell infiltration, usually seen in the walls of true aneurysms [1,13,14], is not observed in that of BBAs [4]. Based on these characteristics, BBAs are considered pseudoaneurysms. Some of them enlarge from a small bulge to a saccular aneurysm in a short time, with a high risk of rebleeding or re-rupture [3,5,15,16]. Despite the theoretical basis for histopathological diagnosis, such as evidence of degenerated internal elastic lamina and abnormal adventitia, obtaining histological samples is not always practical. Imaging findings of BBAs can be subtle, often presenting a slight bulge or irregularity in the arterial wall, making it difficult to detect on initial imaging. Consequently, the radiological findings on conventional vascular imaging (CTA, MRA, DSA) are sometimes indeterminate in BBAs. Therefore, the most reliable current diagnosis is through intraoperative observation [9], wherein the fragile aneurysmal wall can be discerned. Recognizing the need for less invasive imaging-based diagnostic methods, MRI-VWI has been developed, offering a non-invasive solution to improve the preoperative identification of BBAs [7,17-19].

The technique of VWI with a voxel size of less than 1 mm^3^ is particularly advantageous for detecting the characteristic minute vascular changes of BBAs, which are often undetectable with conventional imaging methods [20,21]. Incorporating the black-blood imaging technique into VWI is critical in distinguishing BBAs from the blood signal within the lumen. By suppressing the blood signal, this technique allows for increased contrast between the aneurysm and the surrounding tissues [20,21]. This allows for a clearer visualization of the BBAs, which may present with high signal intensity or contrast enhancement due to the dissection and associated hemorrhage, facilitating easier detection and characterization of the aneurysm. The application of a 3D T1-weighted image provides detailed observation of the vessel wall, offering a 3D perspective that is crucial for assessing the complex anatomy of BBAs. This imaging sequence can provide valuable information about the condition of the vessel wall, including the presence of atherosclerotic plaque, inflammation, or intramural hematoma, which are essential factors in the comprehensive assessment and management of BBAs [20,21]. Prior studies have demonstrated that VWI can reveal contrast enhancement in ruptured cerebral aneurysms [19] and dissecting vertebral arteries [22], suggesting active inflammation or recent hemorrhage within the vessel wall. Our findings are consistent with those of the previous studies, demonstrating that wall contrast enhancement on VWI can serve as a valuable adjunct in localizing the rupture site of BBAs [7]. The utility of VWI in identifying the rupture site in BBAs has not been established. To our knowledge, only a few reports have described the diagnostic usefulness of BBAs [7,23,24]. This case report highlighted the usefulness of VWI for precise and accurate diagnosis of BBAs.

Some limitations of VWI must be acknowledged to optimize its use. First, VWI is susceptible to patient motion, which may be significant in individuals with SAH caused by BBAs. The restless nature of these patients may necessitate sedation during imaging, which in turn requires close monitoring due to the critical state of SAH, thus complicating the imaging procedure. Additionally, the acquisition of high-resolution 3D T1-weighted images is time-consuming. This prolonged imaging time not only exacerbates the challenge of patient motion but may also limit the technique's applicability in urgent clinical scenarios where rapid diagnosis is essential. To address these limitations, innovative solutions have been developed. Regarding the issue of motion sensitivity, a shift from cartesian to radial k-space filling can mitigate the effects of patient motion. This approach allows for motion to be effectively canceled out, thus maintaining image clarity despite patient restlessness. We have already used Compressed SENSE to reduce imaging time. Recently, artificial intelligence-based reconstruction techniques have emerged to further reduce scan time while preserving the signal-to-noise ratio [25]. Furthermore, these methods can be implemented in combination with radial k-space filling [26]. By integrating artificial intelligence-based reconstruction methods into the VWI protocol, the imaging duration can be significantly decreased, which will not only alleviate the difficulties associated with lengthy scan times but also enhance patient comfort and throughput.

## Conclusions

The integration of VWI into the diagnostic workflow can be instrumental in identifying fragile walls of aneurysms such as BBAs more precisely, thereby improving the timing of diagnosis and potentially reducing patient mortality. Moreover, 3D high-resolution VWI helps accurate localization of the rupture site, which is paramount in optimizing treatment strategies.
